# Effect of soybean (*Glycine max* (L.) Merr.) flour inclusion on the nutritional properties and consumer preference of fritters for improved household nutrition

**DOI:** 10.1002/fsn3.751

**Published:** 2018-08-13

**Authors:** Emmanuel Oladeji Alamu, Ibironke Popoola, Busie Maziya‐Dixon

**Affiliations:** ^1^ Food and Nutrition Sciences Laboratory International Institute of Tropical Agriculture (IITA) Ibadan Nigeria; ^2^ Department of Agricultural, Food and Nutritional Science University of Alberta Edmonton Alberta Canada

**Keywords:** consumption intent, protein, proximate composition, sensory properties, soy fritters

## Abstract

Diets in populations of most developing countries are often deficient in protein, carbohydrates, and fat, leading to protein‐energy malnutrition (PEM). Diet‐based strategies are the most promising approach for a sustainable control of PEM. This study aimed to investigate the effects of soy flour inclusion on the nutritional properties, consumer preference, purchase intent, and willingness to pay for wheat‐based fritters. The proximate composition of both types of fritters was determined using standard methods, Consumer preference survey on organoleptic properties was carried out among 291 participants (93 men, 198 women) in Chipata, Katete, and Lundazi districts of Eastern Zambia. The soy‐fortified fritters had significantly higher (*p* < 0.05) levels of ash, fat, amylose, crude fiber, and protein than the unfortified fritters. Protein, crude fiber, amylose, and ash contents of soy‐fortified fritters were considerably increased by 55.5%, 18.9%, 98%, and 30.6%, respectively. The overall preference showed no significant difference (*p* > 0.05) between unfortified and soy‐fortified fritters. A larger percentage of participants in Katete (38%) and Chipata (41%) preferred the soy‐fortified fritters to the nonfortified one. In addition, no significant difference (*p* > 0.05) was also observed for intention to purchase between both types of fritters across the three locations. In conclusion, incorporating 20% soybean flour into fritters, which showed better nutrients quality, could be used to alleviate PEM among fritters consuming populations of developing countries, particularly in Sub‐Saharan Africa.

## 1 INTRODUCTION

According to the Zambian demographic and health survey (ZDHS 2015), 40% of children under the age of five are stunted, 5% wasted, 15% underweight, and 9% of children estimated to be overweight. At 40% stunting rates, Zambia's malnutrition levels are among the highest in the world (Mukuka & Mofu, 2016). The Zambia Demographic and Health Survey (ZDHS) (2015) also revealed that 43.3% of the children in Eastern Province are stunted while 17.4% are severely stunted. Moreover, it has been reported by Centers for Disease Control and Prevention (2013) that protein‐energy malnutrition (5%) has been ranked to be the 4th major cause of death in Zambia, only preceded by HIV/AIDs (20%), Malaria (12%), and lower respiratory infections (7%). Zambia, and Eastern Province, has one of the highest levels of malnutrition in the world with 40% of the children having stunted growth. In addition, Eastern Province has one of the highest rural poverty rates, approximately 78% and this rate is 17% above the country's average (Kuhlgatz & Mofya‐Mukuka, 2015).

Soya bean is a good source of protein and full of beneficial bioactive compounds such as minerals and fat‐soluble vitamins (Alabi & Anuonye, 2007; Hegazy & Ibrahim, 2009; Serrem, Kock, & Taylor, 2011) and has good organoleptic characteristics. Soya bean production in Zambia was approximately 17,500 metric tonnes in 2014 (Central Statistical Office/Ministry of Agriculture (CSO/MAL), 2013‐2014), and Eastern Province ranks fifth in the production. Soya bean protein has been widely used to provide desirable functionalities in many food products at lower cost (Lusas & Rhee, 1995) and has played an important role in human nutrition, particularly in rural households (Dixit et al., 2011; Riaz, 2001).

However, the consumption of soy bean products by Zambian population is minimal, especially in the rural communities, about 70% of soya beans produced in Eastern Province is sold and only 30% is retained at household primarily as seed (Lubungu, Burke, & Sitko, 2013) This is partly due to overdependence on maize as the main staple food and minimum awareness on the nutritional benefits of soya bean products. This high reliance on maize has led to low nutritional standards in the rural communities especially among children.

Food‐to‐food fortification of plant‐based complementary foods has been reported to be an effective strategy for alleviating childhood malnutrition in developing countries (Lartey et al., 1999). In some African countries, soya bean has been exploited for the production of various food products such as, soybean‐fortified *gari* and tapioca (Kolapo & Sanni, 2009), cereal‐based traditional weaning food (Osundahunsi & Awor, 2003), soy–coconut milk‐based yoghurt (Olubamiwa & Kolapo, 2008a), soy–cow milk‐based yoghurt (Olubamiwa & Kolapo, 2008b), and soy–corn milk (Kolapo & Oladimeji, 2008). In Zambia, the few available soy‐processors, such as Community Markets for Conservation (COMACO) processes the soya bean into products such as yummy soy as well as cake and crude oil as by‐products. Approximately 70% of the yummy soy is sold to supermarkets such as Shoprite, Spar, Pick‐n‐Pay, and Melissa, while about 30% is sold to nongovernmental organizations and government health programs such as World Food Program (WFP) and Catholic Relief Service (CRS (Lubungu et al., 2013). Nonetheless, soy‐fortified products have been found to be an alien to most Zambians. Hence, exploring this approach by fortifying soy bean flour with wheat products that Zambian are already accustomed to would be an efficient and cost‐effective approach to alleviating protein‐energy malnutrition (PEM) in Zambia. One such product is fritters, and it is 100% made from wheat flour and is widely consumed snack foods in Zambia, especially in Eastern Province, where there is prevalence of PEM.

Soybean being a very good source of protein and other nutrients and using it to fortify commonly consumed fritters will go a long way in improving nutritional status of rural communities and the Zambian population in general. Therefore, the objective of this study was to compare the nutritional properties, sensory attributes, and willingness to consume nonfortified fritters against fritters fortified with 20% soy flour. However, due to high prevalence of malnutrition despite the high agricultural productivity, the study was conducted in Chipata, Katete, and Lundazi regions of Eastern Province of Zambia.

## 2 MATERIALS AND METHODS

### 2.1 Materials

Wheat flour from hard wheat (*Triticum aestivum*) was purchased from supermarket in Chipata, Zambia, while soybean grains were obtained from the International Institute of Tropical Agriculture (IITA), Zambia.

### 2.2 Processing of soy bean grains into flour

Soy bean grains were processed into flour using a modification of the method described by Alamu, Maziya‐Dixon, Popoola, Gondwe, and Chikoye (2016). Grains were cleaned and sorted to remove stones and other impurities before roasting slightly under low heat until seed coat can be removed by hand. The roasted grains were then coarse‐milled and winnowed to remove seed coat. The decorticated grains were finely milled to 0.5 mm particle size using laboratory mill (Perten, Hägersten, Sweden).

### 2.3 Fritters preparation

Two variants of fritters were made. The unfortified variant was made using 100% wheat flour while the soy‐fortified variant was made by 20% soy flour substitution. The wheat flour is 80% while the soy flour is 20%. This level was chosen because past studies have shown that legumes can be used to supplement cereals at this level without off‐flavor (Alamu et al., 2016).

For the soy‐fortified fritters, the wheat flour (80%) was thoroughly mixed with the soy flour (20%) with the aid of a mechanical mixer before the addition of other ingredients as shown in Table 1. Water was then added to form a sticky dough which was molded into small round pieces of average size of 22 g and deep fried in “Ole oil” (sunflower) obtained from the market. When the fritters were added to hot oil (350–375°F), its surface dehydrates and through a series of Maillard reactions, sugars and proteins breakdown to create a complex flavor and a golden‐brown coloration. It took about 3–5 min to be performed depending on the degree of brownness desired. The weight of the fritters decreased after frying due to dehydration to average size of 20 g.

**Table 1:** Recipes for fritters production

Ingredient	Quantity	
	Unfortified fritters (g)	Soy‐fortified fritters (g)
Wheat flour	1,000	800
Water	800	800
Soy flour	0	200
Baking powder	12	12
Sugar	80	80
Salt	3	3

### 2.4 Proximate composition analyses

Both unfortified and soy‐fortified fritters samples were analyzed for moisture, ash, protein, fat, total sugars, starch, amylose, and crude fiber contents using methods as described by (Alamu, Maziya‐Dixon, Menkir, Olaofe, & Irondi, 2015; AOAC, 2005). All the chemical analyses were performed in duplicate.

### 2.5 Consumer preferences

A total of 291 consumers evaluated the unfortified and soy‐fortified fritters samples. These data were collected from Chipata, Katete, and Lundazi districts of Eastern Zambia. A systematic sampling procedure was used to select respondents for this consumer test. Within each district, the panelists were prescreened, and only those who indicate that they consumed fritters were invited to participate. The panelists' participation in the study was voluntary with informed consent requested from each of them. After panelists' selection, the test was conducted using structured questionnaire designed in English and translated into two local languages, namely “Nyanja” and “Tumbuka.”

The sensory evaluation of the two fritters was carried out according to the methods described by Herbert, Rebecca, and Heather (2012) and Alamu, Maziya‐Dixon, Menkir & Olaofe (2014). The samples were coded with three‐digit random numbers and presented to the participants in a randomised order so that some received the soy fortified first while the other group received the unfortified fritters. This was performed to minimize positional error. Participants were asked to evaluate and rate their preference for crumb color, crumb appearance, aroma, texture, and taste of each sample on 7‐point hedonic scale ranging from “1 = dislike very much” to “7 = like very much.” The results for each quality parameter are expressed as an average of the quality scores from all the panellists. Besides, consumption intent was measured on a 5‐point scale ranging from “1 = will definitely not consume” to “5 = will definitely consume.” Also, respondents were asked to indicate which of the samples they preferred most overall and how much they will be willing to pay for one pack of 100 g of each sample.

### 2.6 Data analysis

Statistical analysis was performed using IBM SPSS statistical software (Version 21). The preference and willingness to consume data were subjected to Analysis of Variance (ANOVA) at 95% level of significance. The overall preference data were subjected to frequency count and chi‐square analysis.

## 3 RESULTS AND DISCUSSION

### 3.1 Proximate analysis of unfortified and fortified fritters

The results of proximate analysis of unfortified and soy‐fortified fritters are shown in Table 2. The soy‐fortified fritters had significantly higher (*p* < 0.05) levels of ash, fat, amylose, crude fiber, and protein than the unfortified fritters. It is obvious from the results that with the addition of soy flour, the ash (30.6%), amylose (98%), protein (55.5%), and crude fiber (18.9%) contents were improved significantly with respect to the fortification except starch content that showed a decrease (50%). As soybean is a rich source of essential nutrients, it can be easily incorporated in the diet to promote desirable health effects (Klein, Perry, & Adair, 1995). The significantly higher levels of protein in soy‐fortified fritters will improve the protein malnutrition status of Zambian consumers. With reference to the Institute of Medicine (IoM) recommended dietary allowance (RDA), consumption of one serving of 100 g/serving of these soy‐fortified fritters product will be enough to satisfy 30% of RDA of 71 g of protein for pregnant and lactating women, one to one‐and‐half serving of soy fritters will satisfy with 100% of RDA of 19–34 g protein for school‐age children, one serving of soy fritters for teenage girls and boys will satisfy 50% of RDA of 46–52 g protein, and one serving of soy fritters for adult men will satisfy 45% RDA of 56 g protein. In addition, there was improvement in amylose and crude fiber contents of soy‐fortified fritters. This will be beneficial to consumer since recently, interest in the utilization of dietary fiber has been increased owing to its relationship with the reduction in blood cholesterol, colon cancer, diabetes, and gall bladder diseases (Newman, Betschart, Newman, & Hofer, 1992).

**Table 2:** Effect of soybean‐fortification on proximate composition of fritters

Chemical parameters	Values (%)	
	Unfortified fritters	Soy‐fortified fritters
Moisture	44.95 ± 0.64^a^	42.01 ± 0.45^a^
Ash	0.86 ± 0.02^a^	1.24 ± 0.02^b^
Fat	0.28 ± 0.20^a^	1.36 ± 0.31^b^
Amylose	0.04 ± 0.12^a^	2.05 ± 0.12^b^
Sugar	10.73 ± 0.06^a^	7.08 ± 0.03^b^
Starch	50.10 ± 0.00^a^	25.13 ± 0.14^b^
Protein	10.35 ± 0.56^a^	23.24 ± 0.33^b^
Crude fiber	5.51 ± 0.14^a^	6.79 ± 0.25^b^

Notes

Value is the mean ± standard deviation.

Values within a row with different letters are significantly different (*p* < 0.05).

### 3.2 Sensory properties of fortified and unfortified fritters

A total of 291 (93 men and 198 women) respondents have participated in the survey across the selected three locations. Respondents' age ranged between 16 and 79 years (mean age 37 years) and close to 80% of the respondents are frequent consumers of fritters (Table 3). This implies that the respondents used for this study covered both young and adults. The effect of soy substitution on sensory attributes ratings of fritters is shown in Table 4 and 5. In terms of appearance and color, the respondents across the three locations found a significant difference (*p* < 0.05) of both fritters samples. Participants preferred the appearance and color of the unfortified fritters due to the lighter color when compared to fortified samples. Similar preference results were observed in a survey conducted on cookies‐fortified soy flour versus the 100% wheat cookies (Ndife, Kida, & Fagbemi, 2014). The development of deeper brown color in fortified fritters could be due to the roasting of the soy grains before milling into flour and possibly Maillard reaction. In addition, it could be due to the presence of sugars and free amino acids, intermediate moisture content, temperature over 50°C, and long processing times could produce brown products such as Amadori compounds and their derivatives to be formed (Mannay & Shadaksharaswany, 2005; Potter & Hotchkiss, 2006). Although there was no significant difference (*p* > 0.05) in preference for taste (F [2, 577] = 2.77) and texture (F [2, 577] = 3.52), respondents in Katete preferred the texture of soy‐fortified fritters to unfortified/fritters. Another study by Rita and Sophia (2010) revealed that there was no significant difference between the taste of whole wheat bread and soy‐fortified bread with up to 30% soy flour as is the case with this study. Moreover, Mohamed, Rayas‐Duarte, Shogren, and Sessa (2006) and Nilufer‐Erdil, Serventi, Boyacioglu, and Vodovotz (2012) mentioned that soy flours can increase firmness and density of bread due to several factors such as dilution of the gluten matrix, formation of defects in the gluten from soy fiber, interchange of disulfide bonds between soy and gluten proteins from wheat flours, and absorption of water by soy fiber causing an increase in dough viscosity. Furthermore, heat processing might produce harder product texture due to protein aggregation and a corresponding loss of protein solubility, as well as water loss (Ribotta et al., 2004). Concerning the overall preference, the frequency count and chi‐square test showed no significant difference between unfortified and soy‐fortified fritters (χ^2^[2, 268] = 4.46, *p* = 0.108). A larger percentage of participants in Katete (38%) and Chipata (41%) prefer soy‐fortified fritters over the unfortified; however, the difference is not statistically significant (Table 6). The intention to consume the two types of fritters was high (mean values above 4 on a 5‐point scale) regardless of whether the fritters was fortified with soy or not. The consumption intent and willingness to pay across the three locations were not statistically significant (*p* > 0.5) for both fritters samples (Table 5), although participants were willing to pay more for the unfortified/plain fritters. This implies that the respondents were ready to consume and willing to pay for soy‐fortified fritters and it could be easily be an alternative to unfortified fritters (Table 7).

**Table 3:** Respondents' characteristics across the three locations

Variables	Location			
	Lundazi (*n* = 101)	Katete (*n* = 90)	Chipata (*n* = 100)	Total (*n* = 291)
Gender				
Male	28 (72.3%)^a^	31 (34.4%)	34 (34.0%)	93 (31.96%)
Female	73 (27.7%)	59 (65.6%)	66 (66.0%)	198 (68.04%)
Age^b^	40 ± 11.1	40 ± 13.8	31 ± 11.9	37 ± 13.0
Minimum	16	18	16	16
Maximum	68	79	79	79
Consumption frequency				
Not at all	9 (8.9%)	—	2 (2.0%)	11 (3.9%)
Rarely	11 (10.9%)	8 (9.0%)	30 (30.0%)	49 (17.1%)
Often	80 (79.2%)	81 (90.0%)	65 (65.0%)	226(79.0%)

Notes

a Incidence number is represented by the number of respondents/total respondent in a particular category.

b Value is the mean ± standard deviation.

**Table 4:** Effects of soy‐fortification on sensory attributes ratings of fritters across all locations

Characteristics	Unfortified fritters	Soy‐fortified fritters
Appearance	6.36 ± 1.03^a^ a	5.94 ± 1.97^b^
Color	6.29 ± 1.14^a^	5.97 ± 1.52^b^
Aroma	6.24 ± 1.22^a^	5.93 ± 1.64^b^
Texture	6.11 ± 1.35^a^	5.86 ± 1.56^b^
Taste	6.33 ± 1.11^a^	6.15 ± 1.39^a^

Notes

Value is the mean ± standard deviation.

Values within a row with different letters are significantly different (*p* < 0.05).

a 7‐point hedonic scale in which 1 = dislike extremely and 7 = like extremely

**Table 5:** Effects of soy‐fortification on sensory attributes ratings of fritters by location

	Unfortified			Soy fortified		
	Lundazi	Katete	Chipata	Lundazi	Katete	Chipata
	Mean (*SD*)	Mean (*SD*)	Mean (*SD*)	Mean (*SD*)	Mean (*SD*)	Mean (*SD*)
Appearance	6.63^{a}d^ (1.00)	6.23^{b}d^ (1.00)	6.22^{b}d^ (1.06)	6.60^{a}e^ (0.85)	5.46^{b}e^ (1.71)	5.75^{b}e^ (1.72)
Color	6.61^{a}d^ (1.03)	6.10^{b}d^ (1.08)	6.15^{c}d^ (1.23)	6.52^{a}e^ (1.05)	5.42^{b}e^ (1.68)	5.95^{c}e^ (1.51)
Aroma	6.56^{a}d^ (1.00)	6.03^{b}d^ (1.33)	6.14^{b}d^ (1.25)	6.36^{a}e^ (1.27)	5.77^{b}e^ (1.66)	5.69^{b}e^ (1.81)
Texture	6.44^{a}d^ (1.22)	5.64^{b}d^ (1.59)	6.19^{b}d^ (1.12)	6.27^{a}d^ (1.39)	5.87^{b}d^ (1.33)	5.47^{b}d^ (1.75)
Taste	6.67^{a}d^ (0.96)	6.17^{b}d^ (1.19)	6.13^{b}d^ (1.12)	6.45^{a}d^ (1.15)	6.18^{b}d^ (1.22)	5.83^{b}d^ (1.68)

Notes

Superscripts within parenthesis {} shows effect of location.

Superscripts outside parenthesis {} shows effect of fortification.

Similar superscripts on the same row are not statistically significant at 0.05.

Different superscripts on the same row are statistically significant at 0.05.

**Table 6:** Overall preference for unfortified and soy‐fortified fritters by location

	Unfortified		Soy fortified		χ^2^
	Frequency	Percent	Frequency	Percent	
Lundazi	40	31	29	20.9	4.46
Katete	37	28.7	53	38.1	
Chipata	52	40.3	57	41.0	

**Table 7:** Effects of soy‐fortification on consumption intent and willingness to pay (WTP) of fritters by location

Location	Unfortified fritters		Soy‐fortified fritters	
	Consumption intent	Mean WTP (Kwacha/20 g)	Consumption intent	Mean WTP (Kwacha/20 g)
Lundazi	4.61 ± 0.88^a^	0.82 ± 0.87	4.46 ± 0.78^a^	0.68 ± 0.39
Katete	4.44 ± 0.74^a^	0.76 ± 0.64	4.42 ± 0.85^a^	0.73 ± 0.60
Chipata	4.33 ± 0.93^a^	0.71 ± 0.42	4.11 ± 1.10^a^	0.84 ± 0.76

Notes

Value is the mean ± standard deviation.

Values within a column with different letters are not significantly different (*p* ≥ 0.05).

## 4 CONCLUSION

The objective of this study was to investigate the effects of soy substitution on nutritional properties, sensory attributes and consumers' willingness to consume and WTP for fritters. It could be concluded that there was a significant increase across all nutritional properties evaluated, especially protein and fiber contents for fortified fritters. In addition, consumer preference results also indicated that both unfortified and 20% soy‐fortified fritters were accepted by the respondents. Although the sensory ratings showed some mild level of preference for some of the attributes of the unfortified fritters, however, overall preference was not significantly different between the fortified and unfortified fritters. Consequently, soy‐fortified fritters could be one of the most alternative options available to supply products with high content of protein as a sustainable solution to public nutrition problems. Considering the dual necessity to enhance the nutritional values and acceptability of soy‐fortified fritters, there is a need to promote and enlighten producers and consumers on the nutritional benefits of soy inclusion. Finally, the findings from this study will contribute to food science and nutrition research and practice, specifically food‐to‐food fortification of plant‐based foods in addressing poor household nutrition and childhood malnutrition in developing countries.

## CONFLICT OF INTEREST

We hereby declare that there is no conflict of interest among authors of this manuscript.
